# Precision medicine for risk prediction of oral complications of cancer therapy–The example of oral mucositis in patients receiving radiation therapy for cancers of the head and neck

**DOI:** 10.3389/froh.2022.917860

**Published:** 2022-08-18

**Authors:** Stephen T. Sonis

**Affiliations:** ^1^Divisions of Oral Medicine, Brigham and Women's Hospital and the Dana-Farber Cancer Institute, Boston, MA, United States; ^2^Department of Oral Medicine, Infection and Immunity, Harvard School of Dental Medicine, Boston, MA, United States; ^3^Primary Endpoint Solutions, Waltham, MA, United States

**Keywords:** precision medicine, oral mucositis, radiation, head and neck cancer, treatment complications

## Abstract

Oral complications of cancer therapy are common, markedly symptomatic, negatively impact patients' quality of life, and add significantly to the cost of care. Patients' risk of treatment-related toxicities is not uniform; most patients suffer at least one side effect, while others tolerate treatment without any. Understanding those factors which impact risk provides opportunities to customize cancer treatment plans to optimize tumor kill and minimize regimen-related toxicities. Oral mucositis (OM) is an iconic example of a clinically significant and common complication of head and neck radiotherapy. Individuals' OM risk is governed by the cumulative impact of factors related to treatment, the tumor, and the patient. In addition to OM risk prediction, a second opportunity to apply precision medicine will evolve as viable treatment options become available. Patients vary widely in how well or poorly they respond to specific treatments. What works well in one individual, might fail in another. Prospective determination of the likelihood of a patient's response or non-response is based on a range of biological interactions. Coupled with risk determination, the application of precision medicine will allow caregivers, patients, and payers to integrate risk/benefit to optimize the probability that the best treatment is be given to the most appropriate patients.

## Introduction

Radiation therapy is a mainstay in the management and treatment of cancers of the mouth and oropharynx and may be used as definitive treatment, following induction chemotherapy, or after surgical resection. Optimally, radiation is administered with concomitant radiosensitizing chemotherapy. Based on current guidelines, cisplatin is the agent of choice. A typical regimen consists of daily (weekdays) fractions of 2 Gy for a total dose of 70 Gy with cisplatin infused either weekly (40 mg/m^2^) or tri-weekly (100 mg/m^2^) [1].

Almost all patients treated with standard regimens of chemoradiation (CRT) for oral or oropharyngeal cancers develop some level of tissue damage in the form of oral mucositis (OM) [2]. The clinical presentation of OM ranges from mild erythema and atrophy to deep, confluent, irregular full-thickness ulcerations impacting the movable oral mucosa. The symptoms, systemic impact and disease burden are proportional to the extent and severity of lesions [3]. While mild manifestations may result in soreness like a food burn, more extensive mucositis is excruciatingly painful and of such intensity that it is refractory to opioids and functionally compromising. Secondary bacterial colonization of ulcerated areas may be associated episodes of bacteremia. In addition, severe mucositis (SOM; defined as ulcerations extensive enough to limit diet to non-solids; WHO grades 3 or 4) may cause treatment breaks which impact negatively tumor response [4]. Patients with SOM visit emergency rooms more often, have more unplanned office visits, are hospitalized more frequently, and are more reliant on parenteral nutrition (gastrostomy feeding) than are patients who have little or no mucositis [5, 6]. It is not surprising that the incremental cost of SOM is over $30,000 (US) [7].

## Opportunities for precision medicine

The application of precision to cancer regimen-related toxicities, including OM, presents two significant opportunities: first, the determination of mucositis risk, and second, assessment of an individual's likelihood of responding to a specific preventive or treatment intervention. Ideally, the probability of both would be determined prior to the initiation of cancer therapy and guide clinicians' and patients' decision-making.

It is clear that there is a risk spectrum for CRT-associated OM. While the majority of patients suffer some level of mucosal damage, a very few complete treatments free of the condition. More commonly are a group of patients (about 30–35%) who develop mild forms of the condition, and a reciprocal cohort (roughly 20%) who manifest the most severe manifestations of OM [8]. Knowledge of a patient's OM risk could be of value in customizing a treatment plan which optimizes tumor control, but limits side effects.

The second opportunity for precision relates to the prediction of who will or who will not respond to a particular treatment. The success of current population-based clinical trials is determined by results associated with the whole study population, even though we know that patients respond to therapies in non-equivalent ways. The dependence of such bell-shaped curve data ignores those patients at either end – those who are hyper-responders or those who do not respond at all. Being able to prospectively identify into which category a particular patient falls informs providers as to dosing decisions or whether to even expose the patient to the proposed treatment [9]. For example, an analgesic at one dose might be very effective in one individual and useless in another. While one patient might benefit from palifermin or another from photobiomodulation, others may not benefit at all. Our ability to differentiate responders from non-responders not only optimizes outcomes for patients, but it saves costs associated with the treatment of non-responders. Treatment should not be a “one size, fits all” proposition. And this approach is true for all forms of treatment – drugs, biologicals, and devices.

## Factors impacting risk definition and outcome assessment

### Defining mucositis as a phenotype

Before trying to understand factors, which contribute to OM risk, it seems critical establish a “gold standard” that defines OM severity. While a patient might be considered to be at risk of SOM when evaluated with one scale, the same risk factor could seem insignificant if SOM is defined by different criteria. Likewise, treatment success defined by one scale might not be observed using another.

Scoring scales for mucositis range from some that are heavily anchored on clinical findings (erythema, atrophy, ulceration, bleeding) to others which categorize severity purely by function (ability to eat a normal diet, soft solids, etc.), or patient-reported symptom-based endpoints [10]. While most studies are interested in “severe” mucositis, this definition is not uniform. For example, WHO scale grades of 3 or 4 (Table 1) are typically defined as severe and mandate the presence of mucosal ulceration, the extent of which is enough to cause the patient to modify the diet to liquids or nothing by mouth. The newest version of the NCI-CTC does not require clinical assessment and relies symptoms and diet, whereas older CTC versions were dependent on clinical descriptors of ulcerations. Even more complicating are those studies, usually retrospective, in which OM severity is based on an interpretation of clinical notes [2].

**Table 1:** World Health Organization Scale (WHO) for scoring oral mucositis.

**Grade**	**Description**
0	None
1	Erythema and oral soreness
2	Oral ulceration; solid diet tolerated
3	Oral ulceration; liquid diet only
4	Oral ulceration; oral alimentation impossible

*The WHO mucositis scoring scale has been unchanged since its introduction more than 30 years ago and has served a the basis for efficacy definitions for many interventional trials. It assesses OM severity based on a combination of symptoms, clinical findings, and patient functionality (diet modifications based on oral symptoms) [11]*.

The lack of a clinically relevant standardized definition of OM also hinders the interpretation of surrogate measures of mucositis such as biomarkers since their accuracy, specificity and sensitivity are measured against standard scale outcomes. Fortunately, while there is not complete congruity in OM scoring, the three most used scales (WHO, NCI-CTC, RTOG) are reasonably consistent in identifying SOM [11].

Aside from scoring scales that is selected, a clinical meaningful definition of what constitutes risk is essential. The trajectory of OM over the course of CRT is remarkably consistent and exposes patients to 3–5 weeks of SOM (weeks five until 2–3 weeks post radiation) [12, 13]. Should patients who develop 2–3 days of SOM be considered to be at the same risk as who develop 4 weeks of the condition? Is severe mucositis incidence (a binary, yes/no endpoint) as important in defining risk as duration? Binary endpoints like incidence are easy to interpret but lack the same consideration of clinical impactfulness as does SOM duration. For example, while a patient with 7 days of SOM might require G-tube feeding or hospitalization for hydration and pain management, it is unlikely that a single day of SOM would have similar consequences.

### Factors affecting mucositis risk

#### Overview

OM risk prediction has long been of interest. In general, risk factors can be grouped into three categories: (1) Those associated with treatment, (2) Those associated with the patient, and (3) Those associated with the tumor [14].

Treatment-associated risk influencers include radiation intensity and field(s) of exposure, inclusion of concomitant chemotherapy and agent selection, and treatment scheduling. Until the biological consequences of CRT and its impact on OM pathogenesis were described risk was almost exclusively based on factors impacting radiation intensity on the oral mucosa. Patient-related variables evolved with more knowledge about radiobiology and the complex biological cascade that defines the progression of mucositis and include genomics, metabolomics, epigenetics, and microbiomics. Finally, the observation that tumor's biological activity and crosstalk with normal tissue influences toxicity risk has been recently noted.

Conceptually, studies for which oral mucositis risk assessment was the primary outcome have only focused on one element at a time – radiation dose, chemotherapy agent and schedule, genomics, etc. Given OM's biological complexity, this approach is naïve as it assumes a linear and causal relationship between a specific risk element and the development of OM, while largely ignoring the dynamics and interaction of the multiple facets which contribute to risk. Indeed, it is possible that in the case of risk determination, 1 + 1 does not equal 2, but might, if one element catalyzes, accelerates, or promotes another, equal 3 or more. A reductionist approach to risk analysis may provide hints, but it is unlikely to describe the consolidated impact of multiple factors [15].

#### Treatment-related factors which might impact mucositis risk

##### Radiation

The stomatotoxic effects of radiation have been extensively described and are associated with the cumulative dose, daily fraction size and schedule, and field [16]. While the administration of concomitant chemotherapy enhances the tumoricidal effect of radiation [17], it also increases OM risk by a factor >3 [18] and hastens it onset [19].

Intensity modulated radiation therapy (IMRT) is currently preferred as it delivers more tumor-focused radiation thereby effectively reducing the level of cumulative radiation delivered to normal mucosal tissue when delivered in daily 2 Gy fractions. Since tumor response is dependent on both cumulative radiation dose and the time over which it is delivered, attempts at using higher daily radiation doses (daily fractions up to 3.5 Gy) in accelerated fractionation regimens have been suggested [20, 21].

While reported survival impacts vary, the stomatotoxicity of these regimens was significant with SOM of such severity as to be the major reason from breaks in treatment and protraction of overall treatment time [22].

In addition to factors associated with radiation dose, field and schedule, timing of radiation administration has been shown to impact mucositis risk as patients treated early in the day are less likely to develop SOM than patients radiated later [23, 24].

##### Concomitant chemotherapy

As noted, the addition of chemotherapy to a standard radiation regimen favorably affects tumor response, but at an expense of added toxicity, including mucositis. While a range of drugs has been used in this role, cisplatin is the gold standard. The original dosing schedule for cisplatin was 100 mg/m^2^ infused on days 1, 21, and 42 of radiation (q3weeks). In response to a challenging systemic toxicity profile, a more conservative scheme of 40 mg/m^2^ weekly evolved. While controversy exists as to the superiority of one regimen vs. the other relative to tumor management, the weekly regimen is more popular in the United States, especially among patients being treated for oropharyngeal cancers. While some have reported that mucositis (all grades qw 61.2 vs. q3w 87.6%; severe qw 12.1 vs. q3w 34%) was more common and severe with high dose cisplatin [25]. In contrast, no differences in either incidence or intensity of mucositis have been reported in other trials in which the tumor impact of the two regimens was evaluated for both oral/oropharyngeal cancers [26–28]. The results of a recently reported Phase 2 interventional trial in which trained evaluators scored mucositis throughout treatment agrees with that conclusion [8].

Since cisplatin cisplatin may not be tolerated by all patients, radiation plus carboplatin, either as monotherapy or with another agent such as 5-fluorouracil, is an alternative. Neither the rate nor severity of mucositis is significantly different than that observed with cisplatin [29, 30].

While of questionable impact on tumor response, the inclusion of EGFR inhibitor as a component of standard cisplatin concomitant chemotherapy appears to increase the risk of oral mucositis [31].

#### Patient-related factors which impact mucositis risk

Patient-related variables dominate OM risk and while multiple factors contribute to risk, the extent to which each factor affects an individual's risk is not the same from patient to patient. Secondly, while the determination of a patient's OM risk represents the collective impact of multiple factors, it is probable that there is biological crosstalk that amplifies or retards the influence of each.

A relationship between past or current tobacco uses on mucositis risk is unclear. Reports of tobacco smoking having no effect on the rate of acute radiation-associated toxicities including mucositis [32] are contradicted by reports that tobacco use is protective of oral mucositis [33, 34] or that smoking adds the risk of mucositis [35, 36].

Sex has been increasingly studied as impacting regimen-related toxicity risk, particularly amongst patients being treated with chemotherapy. For the most part, females appear to be at higher risk than males [37]. Little data exist relative to sex being a risk factor for mucositis in the head and neck cancer population, and to date, conclusions regarding gender are inconsistent with studies suggesting that sex does not significantly increase risk [38], or that males are more likely to be affected [39].

In the case of HNC patients, the events associated with continuous exposure to fractionated doses of radiation have revealed a repeating biological cascade the is initiated with the production of reactive oxygen species and the activation of the innate immune system, is followed by the activation of transcriptions factors, the expression of multiple genes pathways, and the release of mediators that culminates in apoptosis and necrosis of basal epithelial stem cells, atrophy and ulceration. The obvious opportunities for genes to control and influence of these events have led to a range of candidate gene and mutation studies and genome-wide association studies which have attempted to identify genome-based OM risk factors. With very few exceptions, these studies have used peripheral blood monocytes as sources for RNA, and both blood and saliva for DNA of germline origin. The advantages and shortcomings of these has been previously reviewed [40].

In general, three classes of genes have emerged as being particularly associated with mucositis risk, those associated with oxidative stress [41], inflammation [42, 43], those associated with telomere function regulation and its downstream consequences [44], and DNA repair [45].

While somatic mutations have been studied with respect to tumor behavior, the contribution of a tumor's genome to patient toxicity risk has been overlooked until recently. It now appears that both germline and somatic genomic sources contribute to OM. Sumner et al. reported the association of radiation-induced toxicities, including mucositis, and gene alternations expressed in tumor specimens from thirty-seven patients with HNC. More studies are needed to assess how both gene sets interact to affect risk, particularly given the heterogeneity of somatic genes from tumor to tumor [46].

While there seems little doubt that genomics plays a significant role in risk determination, three important considerations remain: (1) The impact of genes on risk is likely the consequence of collective and collaborative activity between and amongst genes so consequently, the risk impact represents the consequences of a collective effect of multiple genes. (2) There is an absence of large-scale prospective trials to confirm the predictive accuracy of proposed risk genes. (3) The global somatic and germ line gene expression impact and their relationship to each other is still lacking.

In addition to genomics influences on risk, metabolomics, epigenomics and proteomics are important, but have yet to be comprehensively studied.

##### Non-genomic peripheral blood markers

High pre-treatment neutrophil/lymphocyte ratios (NLR) (>5) prior to radiation have been proposed as a predictive factor for acute OM [47] as indicators of an inflammatory state. However, others have found NLR as predictive of late-onset OM [48] or not predictive of OM at all [49].

##### The microbiome

Bacterial colonization of OM ulcerations prolongs lesion resolution by provoking the inflammatory response [50]. Speciation studies have suggested that a range of dysbiotic changes impact the progression and severity of mucositis [51], and that individual variations in the microbiome composition may be associated with variations in OM trajectory. Similar patient-specific dysbiosis has been proposed relative to the susceptibility and course of other diseases. It will be critical to assess the microbiome's impact in the context of multivariate analyses (i.e., neutropenia, sampled site, salivary changes, etc.).

More speculative are studies suggesting that bacteria may play an etiologic role in the development of radiation-induced mucositis [52]. These too often fail to account for other local, systemic, and treatment changes with which HNC patients are impacted. The failure of prophylactic antimicrobial strategies in mitigating or attenuating OM further confuses conclusions relative to the importance of the microbiome as an initiator of OM [53].

## Implications of risk determinants on practice and clinical trials

The complexities of OM pathogenesis and their integration with risk determinants present both opportunities for research and challenges in clinical trial design of interventional agents. Given the range of treatment, tumor, and patient-related variables that impact risk and the uncertainty of the weight of each, trying to assure an even playing field for clinical study populations is a high bar. The interactions between risk factors are not two dimensional, but rather a dynamic multiplex problem in which the impact of specific OM risk factors changes over the course of treatment. For example, not only do patient genes interact with each other, but the genome also affects patients' responses to the microbiome, and that response might be more robust at high cumulative doses of radiation then early in the course of therapy. Analyses of these interactions represents a rich opportunity for research to create a hierarchical risk algorithm for OM in which all risk factors are integrated over time.

In the meantime, real world considerations require the assessment of investigational agents in study populations that are not only large enough to evaluate efficacy outcomes taken together, but also sufficient to stratify data to determine the best target population for intervention. For example, a drug which fails to show activity in an “all-comers” study (all HNC diagnoses), might be efficacious for patients with HPV+ cancers, but not HPV- cancers for radiation doses up to 60 Gy. Importantly, given the multifactorial nature of risk factors and those influencing OM trajectory, small study data risks leading to erroneous, misleading, or marginally broadly applicable conclusions.

## Author contributions

The author confirms being the sole contributor of this work and has approved it for publication.

## Conflict of interest

Author SS was employed by Biomodels, LLC and Primary Endpoint Solutions, LLC. Both companies assist industry, government and academics to study and enable drugs, biologicals and devices to treat patients for a variety of indications including cancer and the side effects and toxicities of its treatment. SS does not have equity in any of the companies with which he works.

## Publisher's note

All claims expressed in this article are solely those of the authors and do not necessarily represent those of their affiliated organizations, or those of the publisher, the editors and the reviewers. Any product that may be evaluated in this article, or claim that may be made by its manufacturer, is not guaranteed or endorsed by the publisher.
